# *Curtobacterium* sp. Genome Sequencing Underlines Plant Growth Promotion-Related Traits

**DOI:** 10.1128/genomeA.00592-14

**Published:** 2014-07-17

**Authors:** Daniela Bulgari, Andrea Minio, Paola Casati, Fabio Quaglino, Massimo Delledonne, Piero A. Bianco

**Affiliations:** a Department of Agricultural and Environmental Sciences—Production, Landscape, Agroenergy, University of Milan, Milan, Italy; b Department of Biotechnology, University of Verona, Verona, Italy

## Abstract

Endophytic bacteria are microorganisms residing in plant tissues without causing disease symptoms. Here, we provide the high-quality genome sequence of *Curtobacterium* sp. strain S6, isolated from grapevine plant. The genome assembly contains 2,759,404 bp in 13 contigs and 2,456 predicted genes.

## GENOME ANNOUNCEMENT

The genus *Curtobacterium* belongs to the family *Microbacteriaceae* and includes a wide range of bacteria isolated from soil, cheese vat, residential carpet, and plants. *Curtobacterium*-related strains were isolated as endophytes from sweet-orange, coffee, grapevine, and poplar (1–4). Some *Curtobacterium*-related bacteria were reported as etiological agents of plant diseases (5), while *Curtobacterium flaccumfaciens* protected cucumber plants from pathogens (6) and led to induced systemic resistance (ISR) in other plant hosts (7). We sequenced the complete genome of *Curtobacterium* sp. strain S6, previously isolated as an endophyte from grapevine plant (4).

*Curtobacterium* sp. strain S6 was cultivated in Luria-Bertani (LB) liquid medium at 37°C overnight. Its genomic DNA was extracted using the GenElute bacterial genomic DNA kit (Sigma-Aldrich), with some modification. DNA libraries were prepared using the TruSeq DNA sample prep kit (Illumina), quality checked by analysis with a Bioanalyzer DNA high-sensitivity kit, quantified by real-time PCR, and sequenced as 100-bp paired-end (X2) reads using an IlluminaHiSeq 1000 system within a single lane. A total of 5.8 Gb with 2,099-fold coverage of the genome was generated from a 400-bp paired-end (100-nucleotide [nt] X2) library. Sequenced reads were preprocessed by removing low-quality reads (undetermined bases >10% total length; >50 bp with quality score of <7), clipping adapters (Scythe 0.980) (https://github.com/vsbuffalo/scythe), trimming low-quality read ends (quality score, <20), and discarding reads with a final length of <20 nt (Sickle 0.940) (https://github.com/najoshi/sickle).

Genome assembly was performed using SOAPdenovo2 software (8). The genome assembly contains 2,759,404 bp in 13 contigs (minimum contig length, 200 bp; maximum contig length, 1,587,751 bp), with an average GC content of 65%. Gene annotation was performed using the RAST server (9), revealing 2,456 predicted protein-coding genes. Genes were functionally annotated using Blast2go software (10). The *Curtobacterium* sp. strain S6 genome was characterized for the presence of beneficial traits related to plant mineral nutrition (phosphate solubilization and siderophores), development (indolacetic acid [IAA] synthesis), stress relief (1-amino-cyclopropane-1-carboxylate [ACC] deaminase and catalase activity), and disease control (chitinase activity and siderophores). *In vitro* assays showed that *Curtobacterium* sp. strain S6 solubilizes phosphate and produces IAA, and it showed catalase and ACC deaminase activity.

Despite its biological significance and its possible involvement in plant defense responses against pathogens (11), complete genome sequence information for the *Curtobacterium* genus is still limited. Thus, our work may lead to *Curtobacterium* genome-based biotechnological applications for developing sustainable biocontrol strategies against plant diseases.

### Nucleotide sequence accession numbers.

This whole-genome shotgun project has been deposited at DDBJ/EMBL/GenBank under the accession number JHEL00000000. The version described in this paper is the first version, JHEL01000000.
